# 

**Published:** 1888-10

**Authors:** 


Vol. IX. OCTOBER, 1888. No. 10.

LIBRARY
INDEXED
S.G.O.

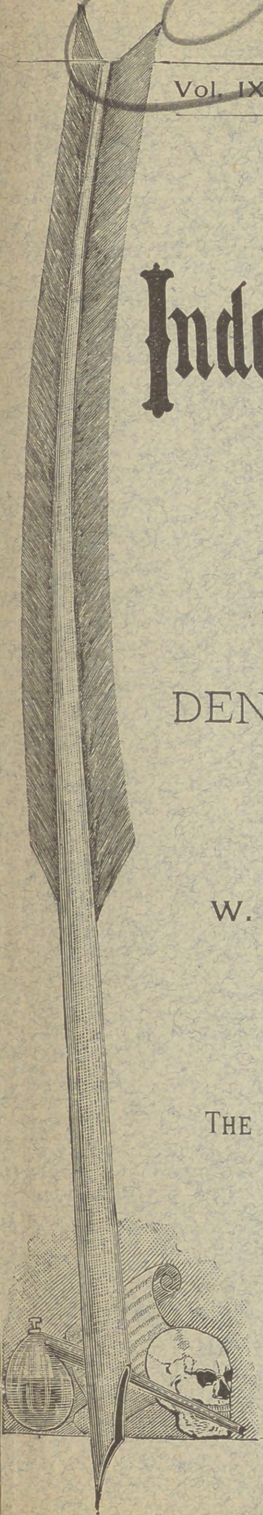

### THE Independent Practitioner:

A MONTHLY JOURNAL

DEVOTED TO

DENTAL AND ORAL SCIENCE.

---

W. XAVIER SUDDUTH, M. D., D. D. S.,

EDITOR.

---

THE INTERNATIONAL DENTAL JOURNAL ASSOCIATION,
PUBLISHERS,
51 West 37th Street, New York City,
AND
1215 Filbert Street, Philadelphia.

$2.50 Per Annum in Advance, . . . . Single Copies, 25 Cents.

Entered at the Post-Office, Philadelphia, Pa., as Second-Class matter.

